# Towards a Greener Approach for Biomass Valorization: Integration of Supercritical Fluid and Deep Eutectic Solvents

**DOI:** 10.3390/antibiotics12061031

**Published:** 2023-06-08

**Authors:** Jelena Vladić, Martina Jakovljević Kovač, Valentina Pavić, Stela Jokić, Siniša Simić, Alexandre Paiva, Igor Jerković, Ana Rita Duarte

**Affiliations:** 1 LAQV/REQUIMTE, Faculdade de Ciências e Tecnologia, Universidade Nova de Lisboa, 2829-516 Caparica, Portugal; abp08838@fct.unl.pt; 2 Faculty of Technology, University of Novi Sad, 21000 Novi Sad, Serbia; sinisa.simic@uns.ac.rs; 3 Faculty of Food Technology, Josip Juraj Strossmayer University of Osijek, 31000 Osijek, Croatia; martina.jakovljevic@ptfos.hr (M.J.K.); sjokic@ptfos.hr (S.J.); 4 Department of Biology, Josip Juraj Strossmayer University Osijek, 31000 Osijek, Croatia; vpavic@biologija.unios.hr; 5 Faculty of Chemistry and Technology, University of Split, 21000 Split, Croatia; igor@ktf-split.hr

**Keywords:** *Lavandula stoechas*, supercritical CO_2_, deep eutectic solvent, antibacterial activity, antioxidant activity

## Abstract

A green and sustainable procedure for obtaining *Lavandula stoechas* extracts with antioxidant and antimicrobial properties was investigated. Green solvents, supercritical CO_2_, and natural deep eutectic solvents (NADES) together with ultrasound-assisted extraction were used for the sequential extraction of terpene and polyphenols fractions. After the CO_2_ extraction of the terpene fraction, the residue material was used in an extraction with different NADES (betaine-ethylene glycol (Bet:EG), betaine-glycerol (Bet:Gly), and glycerol-glucose (Gly:Glu)), intensified with an ultrasound-assisted method (at 30 and 60 °C). In the CO_2_ extract, the major group of components belonged to oxygenated monoterpenes, while the highest polyphenol content with the dominant rutin (438.93 ± 4.60 µg/mL) was determined in Bet:EG extracts (60 °C). Bet:EG extracts also exhibited the most potent antioxidant activity according to DPPH, ABTS, and FRAP assays. Moreover, Bet:EG extracts showed significant inhibitory activity against Gram-positive and Gram-negative bacteria, with minimum inhibitory activity of 0.781–3.125 and 1.563–6.250 mg·mL^−1^, respectively. By comparing the polyphenolic content and antioxidant and antimicrobial activities of Bet:EG extracts with extracts obtained with conventional solvents (water and ethanol), the superiority of NADES was determined. The established environmentally friendly procedure unifies the requirements of green and sustainable development and modern pharmacognosy because it combines the use of safe alternative solvents, the absence of solvent waste generation, more rational use of resources, and the attainment of safe and quality extracts.

## 1. Introduction

Antimicrobial resistance (AMR) is a global concern, which is estimated to threaten the lives of millions of people around the world (by increasing mortality and morbidity). In 2019, the deaths of 4.95 million people were associated with drug-resistant bacterial infections, while 1.27 million deaths were directly caused by AMR [1]. Additionally, foodborne pathogens affect millions of people annually, and many foodborne diseases can result in very serious and fatal consequences. The World Bank estimated that if AMR is not addressed, it would lead to an increase in healthcare costs of up to 25% in low-income countries and 8% globally. Therefore, this situation could push up to 28 million people into poverty by 2050 [2]. Due to such extensive consequences, AMR has significant effects on a range of Sustainable Development Goals (SDG); hence, without overcoming AMR, it is not possible to achieve the SDGs. Multisectoral and coordinated cooperation on the identification of new antimicrobials and the reduction of uncontrolled and excessive use of antibiotics is one of the primary goals in the strategy to combat AMR. Due to their diverse chemical profile and significant antimicrobial potential, medicinal plants can be a promising resource and part of the strategy for overcoming AMR.

Aromatic medicinal plants have a long tradition of use around the world. This presence and the increasing preference of consumers for natural products were the drivers behind the commercial recognition of aromatic medicinal plants. Therefore, there is a large number of products based on medicinal plants present in the market. However, for medicinal plant-based products to remain relevant, it is necessary that research in the field of natural product development keep pace with the needs of modern development and aim to create green innovation and sustainable development, as defined within the SDGs.

Therefore, research in the field of medicinal plants includes (i) the development of products that target global problems such as AMR, (ii) the use of safe green solvents, (iii) the use of renewable materials, (iv) the reduction of harmful waste generation, and (v) the reduced use of energy, in line with the SDGs.

Among the species whose traditional use is very widespread and diverse is *Lavandula stoechas* L., which belongs to the family Lamiaceae and the genus Lavandula. The chemical profile of *L. stoechas*, rich in different classes of compounds, such as terpenes, phenols, flavonoids, and coumarins, is responsible for numerous biological actions, including antioxidant, anti-inflammatory [3], antimicrobial, antispasmodic [4], insecticidal, and larvicidal activities [5].

Isolation of bioactive compounds and production of *L. stoechas* products are most often carried out using conventional extraction methods: terpene fraction hydrodistillation or steam distillation [6,7,8], while the phenolic fraction is obtained by solid–liquid extraction with organic solvents, such as methanol [3,9], aqueous–methanolic mixture [4], and ethanol [5]. The mentioned conventional methods for obtaining essential oil and extracts of *L. stoechas* do not fit into modern production procedures because they do not ensure a safe product profile and a green sustainable production process. Namely, they are characterized by low extraction efficiency and lack of selectivity, as well as a large environmental footprint due to the use of toxic solvents that must be removed from the final product, which increases the cost of the entire process. Therefore, alternative extraction technologies and solvents have been developed which, compared to conventional methods and solvents, are safer to work with and offer the possibility of changing selectivity and adjusting the final quality of the product.

Among alternative solvents, supercritical CO_2_ has emerged as a successful alternative for the extraction of lipophilic components from materials, which ensures obtaining a clean and safe extract of high quality. Due to its characteristics, which include inertness, non-toxicity, mild conditions (31.1 °C and 73.8 bar) to reach the supercritical state, and the possibility of recirculation, it has already been implemented in commercial large-scale production in several applications, including pharmaceutical and food, and industrial-scale biochemical and chemical reactions [10]. Additionally, apart from extraction, it has been shown that the use of supercritical CO_2_ can also play a role in the pretreatment of biomass before further extractions [11,12]. As a result of the exposure of the material to pressurized CO_2_, there is a loosening of the cell walls which facilitates mass transport and the extraction of non-polar components by CO_2_. The hypothesis behind this work is that due to the potential physical change in the structure of the material, the matrix can be more permeable and thus facilitate the subsequent extraction of the polar fraction and achieve a more rational use of the material [13]. It was previously shown that supercritical CO_2_ can be used for the extraction of non-polar components from *L. stoechas* [14,15,16,17]. However, none of the works investigated the possibility of a more efficient use of the material. For this purpose, two green technologies, namely supercritical fluids and natural deep eutectic systems (NADES), were coupled.

NADES represent a new generation of alternative solvents characterized by low cost, easy synthesis, non-flammability, chemical and thermal stability, tunability, and versatility [18]. Due to their low toxicity and natural origin, they are considered environmentally safe and biodegradable solvents. NADES represent a mixture of natural components whose melting point is lower than the melting points of individual components when at a particular molar ratio [19]. They are usually prepared by mixing and stirring the components until a liquid is formed, therefore 100% atom economy is achieved and further purification is not necessary [20].

**Figure 1:**
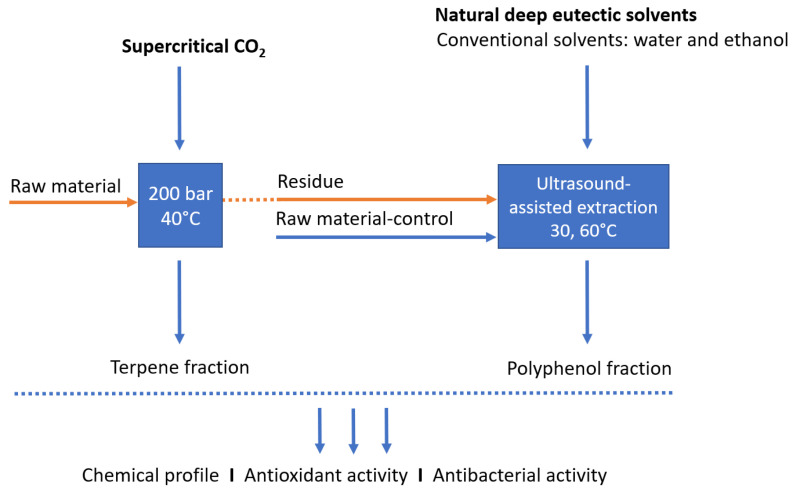
Schematic diagram of the sequential extraction procedure of *Lavandula stoechas*.

This work investigated the sequential extraction of non-polar and polar fractions and more efficient utilization of *L. stoechas* material, using green solvents supercritical CO_2_ and NADES, to obtain potent antioxidant and antimicrobial extracts (Figure 1). In the first step, supercritical CO_2_ extraction was performed for the extraction of lipophilic components, while the exhausted raw material (residue) was used in the next step for the extraction of polar compounds using NADES and ultrasound-assisted extraction. In addition, in order to determine whether the CO_2_ extraction can play the role of pretreatment and help the release of polar components, extraction with NADES was performed with both, the raw material after CO_2_ extraction (residue), and the raw material not subjected to CO_2_ extraction (non-residue; control). The obtained extracts were examined in terms of chemical composition and their potential as antibacterial and antioxidant agents was investigated.

## 2. Results

### 2.1. Chemical Profile of L. stoechas Extract Obtained by Supercritical CO_2_ Extraction

**Figure 2:**
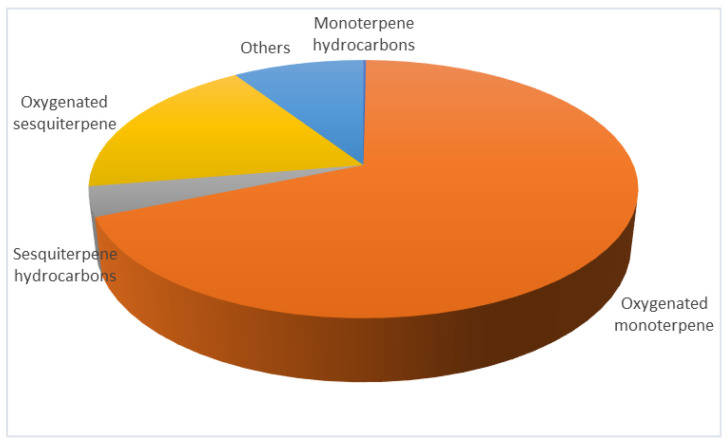
GC/MS analysis of *L. stoechas* supercritical extract (200 bar, 40 °C, and 3 h) [21].

In our previous study based on which the conditions for the supercritical extraction were selected, the obtained extracts were analyzed by the GC-MS method. Therefore, to confirm the chemical composition of the extract obtained in this study (conditions 200 bar, 40 °C, and 3 h), GC-MS was also applied, and the results were similar to the ones reported (Figure 2), where the total extraction yield was 1.99% (*w/w*) [21] In the GC-MS profile of the extract, oxygenated terpenes were the most abundant with 72.21%. Within oxygenated terpenes, monoterpenes were more dominant (55.41%) with major components fenchone (12.93 ± 2.94%) and verbenone (12.08 ± 1.27), followed by camphor (7.80 ± 2.46) and 1,8-cineole (7.51 ± 2.02%). From the group of oxygenated sesquiterpenes, the most represented was α-cadinol 8.31 ± 1.88%, while viridiflorol and ledol were present with 2.64 ± 0.46% and 1.98 ± 1.01%, respectively. Moreover, minor oxygenated terpene compounds characteristic of the species *L. stoechas* were identified, such as borneol, bornyl acetate, lavandulyl acetate, linalool, *α*-cyclogeraniol, and others. Terpene hydrocarbons, monoterpenes 0.76%, and sesquiterpenes 2.61% were significantly less represented. Additionally, non-terpene components such as fatty alcohol and acids, alkanes, carboxylic acids, and esters (9.26%) were identified.

### 2.2. Chemical Profile of L. stoechas Extracts Obtained by Ultrasound-Assisted NADES Extraction

**Figure 3:**
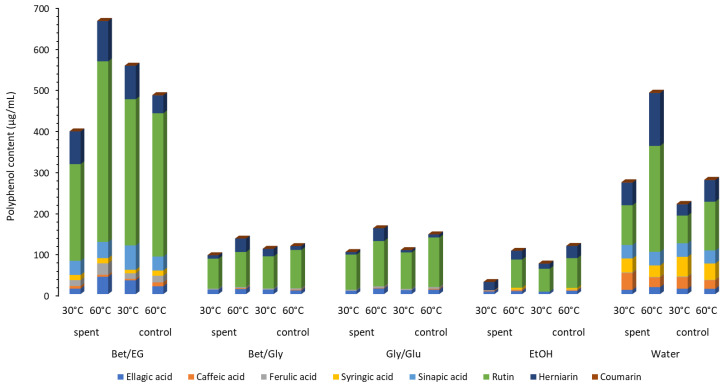
Content of polyphenols in NADES extracts.

After the CO_2_ extraction of the non-polar fraction, the residue material was further used for ultrasonic-assisted NADES extraction, which was performed at 30 and 60 °C for 30 min. In order to establish if the supercritical CO_2_ extraction can intensify the sequential extraction of polar compounds, ultrasound-assisted NADES extraction was also performed with non-residue material (control). Additionally, to evaluate the potential of NADES as solvents, conventional solvents, namely ethanol and water, were used. All obtained NADES and conventional extracts were examined in terms of chemical composition using HPLC analysis (Figure 3; Supplementary Materials, Table S1).

The presence of different classes of components, including flavonoids, hydrolyzable tannins, derivatives of hydroxycinnamic and hydroxybenzoic acids, and coumarins was identified in the extracts. Observing the total sum of identified components (Figure 3, Supplementary Materials, Table S1), the highest phenolic content was recorded in the extracts obtained with Bet:EG (residue, 60 °C) and accounted for 664.19 µg/mL, while the second highest content was in the extract obtained from the extraction with the control material at 30 °C (555.43 µg/mL). Bet:EG extracts proved to be several times richer in polyphenols compared to Bet:Gly and Gly:Glu. Furthermore, ethanol extracts had the lowest polyphenol content, while the water extracts were richer in polyphenols than ethanolic, Bet:Gly, and Gly:Glu extracts. Extraction of non-residue material with Bet:EG and residue material with water (60 °C) had approximate values of 483.78 and 489.80 µg/mL, respectively.

At a higher temperature of 60 °C, a positive effect of the CO_2_ treatment was observed, so NADES and water extracts with a higher polyphenolic content were obtained compared to the control samples. During CO_2_ extraction, the material is exposed to high pressure and saturated with CO_2_, as a result of which there is a weakening of the material structure and disruption of glandular structures where non-polar components are located, and their extraction. At the end of the CO_2_ extraction, depressurization is performed, and there is a sudden drop in pressure which may affect the structure of the plant material. Due to pressure changes, the resistance of the material further weakens, potentially facilitating solvent penetration and access to polar components in a subsequent step. In order to further intensify the NADES extraction, UAE was used, where the pressure is transmitted as a series of compressions and decompressions, resulting in cavitation. As a consequence of collapsing cavitation bubbles, various effects on the material occur, such as fragmentation, pore formation, erosion of the material, shear force, and swelling, which, due to the increase in permeability of the material, facilitate the penetration of solvents into the material and intensifies the extraction [22,23]. The interaction between ultrasonic extraction conditions, solvent, and material characteristics determines the efficiency and selectivity of component extraction. Due to CO_2_ exposure, as well as the effect of UAE, the permeability of the material and surface area increases, and there is more effective solubilization of components in the solvent and improved mass transfer in the boundary layer of solid material. It was previously reported that the use of CO_2_-treated microalga biomass improved the yield of ultrasound-assisted, microwave-assisted, and subcritical water extraction, compared to CO_2_-untreated microalga biomass [13]. Also, the pretreatment with high pressure has been shown to be effective in improving the yield of CO_2_ extraction of *Origanum virens* and *Hypericum perforatum* [11,12].

The higher temperature was more effective in terms of polyphenolic content compared to the lower one at 30 °C. Increasing the temperature increases the solubility and desorption of target components in the solvent, but temperature also affects the characteristics of the solvent, reducing its viscosity and thus improving diffusivity into the material [22]. Additionally, the viscosity of NADES is one of the parameters that can significantly limit extraction. Due to the lowering of the temperature (30 °C), the viscosity increases, and the solvent penetration is aggravated; thus, the interaction with the analyte and its transition into the solvent is reduced. Viscosity values of NADES solvents were Bet:EG 18.27 ± 0.21 (60 °C) and 48.63 ± 0.42 (30 °C); Bet:Gly 227.33 ± 4.66 (60 °C) and 1250.10 ± 0.0028 (30 °C); Gly:Glu 525.37 ± 28.76 (60 °C) and 5443.90 ± 46.53 (30 °C) [24]. Bet:EG proved to be the most efficient NADES for the extraction of polyphenols, which is correlated with the lowest viscosity among the NADES used.

Observing the effect of temperature on the control material, it was noted that increasing the temperature from 30 to 60 °C led to an increase in polyphenolic content in all samples, except for Bet:EG control, where a decrease was observed with increasing temperature. Namely, although the viscosity decreases and penetration of the solvent increases with temperature, weakening of the cavitation effect can also occur. The potential explanation can be that due to the sequential collapse of cavitation bubbles at high temperature, shear stress increases and this can further cause degradation of the target component [22]. Additionally, the decrease in surface tension of the solvent at higher temperatures may lead to a decrease in the intensity of the collapse of cavitation bubbles, which further lowers the mass transfer and extraction efficiency [22].

The flavonoid rutin was the most abundant in extracts in the range from 55.53 µg/mL (ethanol extract, control, 30 °C) to 438.93 ± 4.60 µg/mL (Bet:EG, residue, 60 °C). Moreover, the extract obtained by extracting the CO_2_-treated material with Bet:EG at 60 °C had the highest content of ferulic acid and ellagic acid (27.84 ± 0.60 µg/mL and 42.21 ± 2.63 µg/mL, respectively). Sinapic acid was identified only in Bet:EG and water extracts, while syringic acid was detected in Bet:EG and water extracts, and alcoholic extracts obtained at 60 °C. Significantly higher viscosity values of Bet:Gly and Gly:Glu were the most likely cause of the less efficient extraction of polyphenols.

For the extraction of caffeic acid and syringic acid, the most adequate solvent was water in combination with low temperature (40.64 ± 0.16 µg/mL). Low viscosity, high polarity, and high affinity to caffeic acid were previously established [25]. Additionally, water was the most adequate for the extraction of herniarin at 60 °C.

The polyphenolic profile of *L. stoechas* extracts varies in the literature depending on the applied extraction technique, solvents, and material characteristics. The presence and different presence of polyphenolic components may be due to different stages of development and environmental conditions that dictate the development of different adaptive mechanisms to different environmental conditions, such as air humidity, light, temperature, presence of nutrients, pathogen attacks, and others [26]. Ceylan et al. [9] reported caffeic acid, rutin, and rosmarinic acid as major components of the methanol extract of *L. stoechas* from Turkey. Epicatechin was found to be the major compound in ethyl acetate and acetone extracts, while sinapic acid was the major compound in methanolic and ethanolic extracts [26].

### 2.3. Antioxidant Activity of L. stoechas Extracts

Different tests were used for the analysis of antioxidant activity due to the differences in the properties of the extracts, their constituents, and the possibility of different interactions with the used radicals. Free radical scavenging activity assays DPPH and ABTS, and ferric-reducing antioxidant power FRAP assay were applied. DPPH is based on the color change of the stable violet DPPH radical into yellow diphenyl picryl hydrazine, due to the acceptance of electrons. The ABTS assay is based on the reduction of ABTS+• radicals by antioxidants present in extracts. FRAP method is based on the reduction of a colorless complex Fe^3+^-tripyridyltriazine to a blue-colored complex Fe^2+^-tripyridyltriazine by the action of electron-donating antioxidants.

The supercritical extract showed antioxidant activity measured by DPPH-IC_50_ and ABTS-IC_50_ tests of 2.34 mg/mL and 1.73 ± 0.12 mg/mL, respectively. However, high FRAP-IC_50_ (18.58 ± 3.09 mg/mL) indicated low ferric-reducing ability. Therefore, the antioxidant activity of supercritical extracts was manifested more significantly through the scavenging of free radicals. A significantly lower ferric-reducing capacity of essential oil compared to the extract rich in phenolic components was also observed in the study by Beghlal et al. [27] and was attributed to the greater antioxidant potency of polyphenols. The reducing power of phenol is probably due to the presence of hydroxyl groups, which may serve as electron donors [27]. Additionally, the FRAP test is more adequate for hydrophilic components, while DPPH and FRAP enable the determination of the antioxidant capacity of hydrophilic and lipophilic compounds [28].

By analyzing the antioxidant activity of NADES extracts of *L. stoechas*, the DPPH-IC_50_ values were in the range of 0.25 ± 0.08–5.31 ± 0.99 mg/mL, ABST-IC_50_ in the range of 0.47 ± 0.03–11.14 ± 1.56 mg/mL, and FRAP-EC_50_ varied between 0.52 ± 0.04–8.60 ± 0.88 mg/mL (Table 1). According to all three antioxidative tests, Bet:EG extracts were the most potent among the NADES extracts. Compared to Gly/Glu and Bet:Gly extracts, Bet:EG extracts exhibited approximately 7–10 times stronger antioxidant activity, which corresponds to the significantly lower polyphenol contents in Gly/Glu and Bet:Gly extracts. Gly:Glu extracts showed higher antioxidant activity than Bet:Gly extracts. Considering the effect of the temperature on the IC_50_ value, Bet:EG extracts obtained at 60 °C presented a slightly lower value than those at 30 °C, although not statistically significant. Compared to supercritical extract, Bet:EG demonstrated stronger antioxidant activity. On the other hand, the supercritical extract was on par with Gly/Glu extracts.

Ethanol extracts presented the lowest antioxidant activity, while water extracts showed an antioxidant activity similar to Bet:EG extracts, with no statistically significant differences. The obtained IC_50_ values were similar to the values (0.3 ± 0.010 mg/mL) obtained by Ceylan et al. [9] where the extract was obtained by extraction with methanol at 35 °C using a magnetic stirrer for 6 h (20 g/500 mL).

Pearson correlation test was used to determine the correlation between the antioxidant activity of extracts and the content of polyphenolic components (Table 2). Total phenol content and sinapic acid exhibited a very strong negative correlation with antioxidant activity determined using the DPPH test, with very high statistical significance (*p* < 0.0001). Additionally, herniarin had a strong correlation (*p* < 0.001), while the other components (except coumarin) had a moderate-to-strong negative correlation with antioxidant activity (DPPH) with statistical significance (*p* < 0.05), which means there is a tendency for low IC_50_ (high antioxidant activity) to associate with high polyphenolic scores (and vice versa). The antioxidant capacity of the extracts determined by the other two tests, ABTS and FRAP, had a weaker correlation with the total and individual components compared to the DPPH test.

Therefore, the established correlation clearly indicates that extracts with a higher polyphenolic content exhibited more pronounced DPPH and ABTS scavenging capacities and reducing power, that is, antioxidant activity depended on the quantitative content of polyphenolic components. However, the antioxidant activity was also conditioned by the qualitative aspect of the identified polyphenols, that is, by their individual antioxidant capacity.

In the case of phenolic acids, the antioxidant activity is conditioned by the number and position of hydroxyl groups attached to the aromatic ring of the benzoic or cinnamic acid [29]. For instance, caffeic acid, due to the free phenolic group, has a significantly higher antioxidant activity than ferulic acid, whose antioxidant properties are significantly reduced due to the presence of the methoxyl group. This was confirmed according to different methods and tests, ferric reducing power [29], scavenging the free radical [30], and inhibiting peroxidation of LDL [31]. In our work, the most prominent presence of caffeic acid was determined in the water extract obtained at 30 °C, which corresponded to the lowest measured DPPH-IC_50_ value of 0.25 ± 0.08 mg/mL (DPPH), followed by the samples extracted with Bet:EG.

Syringic acid, derivate benzoic acid, due to the presence of a methoxy group attached to the aromatic ring exhibits considerable antioxidant activity [32]. However, derivatives of cinnamic acids are more active antioxidants than the derivatives of benzoic acid. The presence of the CH=CH–COOH group in cinnamic acid derivatives ensures a greater efficiency than the COOH group in benzoic acids [33]. In our study, a significantly higher content of cinnamic acid derivates in the Bet:EG extracts matched the stronger antioxidant activity of these extracts compared to other NADES extracts.

Ceylan et al. [9] reported that phenolic acids and flavonoids of *L. stoechas*, such as rutin and caffeic acid are responsible for the antioxidant activity and according to Sanchez-Moreno et al. [34] rutin was more potent than ferulic acid and its antioxidant capacity is due to its flavonoid structure. Bet:EG extracts were multifold richer in rutin compared to remaining NADES extracts, which could be the reason behind the more potent antioxidant capacity of these extracts. Furthermore, extracts the most abundant in herniarin, Bet:EG and water extracts, exhibited the strongest antioxidant effect. Herniarin belongs to the class of coumarins and its significant antioxidant activity is related to its benzopyrone structure, which is chemically analogous to flavonoids [35].

However, in most cases, the antioxidant activity is considered to be the result of the synergistic effect of all the components present in the mixture. Hence the cumulative presence of polyphenolic compounds observed in the Bet:EG extracts rendered more powerful antioxidant extracts.

### 2.4. Antibacterial Activity of L. stoechas Extracts

Antibacterial activity of the supercritical extract, NADES extracts, and control extracts (ethanolic and water) was tested against Gram-positive bacteria, *B. subtilis* and *S. aureus*, and Gram-negative bacteria, *E. coli* and *P. aeruginosa* (Table 3).

Supercritical extract showed antibacterial activity, with MIC 3.39 mg·mL^−1^ against *E. coli*, *B. subtilis*, and *S. aureus*, while the least resistant was *P. aeruginosa* with MIC 6.775 mg·mL^−1^. Greater resistance of *P. aeruginosa* to essential oil *L. stoechas* was also reported in the study by Bouyahya et al. [36]. Additionally, the stronger antibacterial activity of L. *stoechas* against Gram-positive bacteria compared with Gram-negative bacteria is in accordance with the literature [36,37,38].

Among NADES extracts, Bet:EG were selected for antibacterial activity testing because they were significantly richer in polyphenols and with more pronounced antioxidant activity compared to other NADES extracts. Compared to the supercritical extract, Bet:EG extracts exhibited a stronger antibacterial activity. Gram-positive bacteria were more susceptible to Bet:EG extracts, so lower MIC values were achieved, 0.781–3.125 mg·mL^−1^, compared to Gram-negative MIC between 1.563 and 6.250 mg·mL^−1^. Bet:EG extracts obtained at 60 °C (residue or non-residue) exhibited stronger antibacterial activity against the pathogens than extracts at 30 °C, which is consistent with the antioxidant activity of these extracts. The most active extract was obtained from the control material at 60 °C, while the activity of the control at 30 °C and residue extract at 60 °C was equal.

No clear correlation was observed between the inhibitory effect on bacteria and the content of polyphenols. At both temperatures, extracts obtained from the control material showed stronger antibacterial activity compared to the residue. The reason may be an additive or synergistic antimicrobial effect due to the presence of other polyphenolic components with antimicrobial activity that were extracted using NADES, but which were not detected in the extracts. The effect of natural extracts is commonly the result of the synergistic effect, where minor compounds can play a very important role in enhancing the antimicrobial effect of the major component [39].

Water extracts exhibited activity against *E. coli* that was equal to Bet:EG extracts (control, 60 °C). However, for the other tested bacteria, Bet:EG samples (control, 60 °C) were more effective than water extracts. Additionally, the activity of water extracts did not depend on either the extraction temperature or the type of raw material used. Ethanol extracts obtained at high temperatures were more effective than water extracts and had the same activity as NADES extracts. Additionally, in the case of ethanol extracts, the type of raw material used had no influence on their activity at both temperatures.

Ez Zoubi et al. [40] investigated the antimicrobial activity of hydro-ethanolic, flavonoid, and tannin *L. stoechas* fractions/extracts against pathogenic bacteria *P. aeruginosa* and *S. aureus*. They determined that the hydro-ethanolic extract exhibited antibacterial activity with a MIC value against *P. aeruginosa* of 40 mg/mL, while it was 80 mg/mL against *E. coli* and *S. aureus*, while the MIC of flavonoid fractions, which showed the highest effect against bacteria, was from 10 to 40 mg/mL. Additionally, testing aqueous and methanol extracts of *L. officinalis* against Gram-positive and Gram-negative pathogenic multiresistant bacterial strains, Al-Niaame and Aziz [41] found that methanol extracts are active towards all bacteria with MICs of 25 mg/mL, while aqueous ones were not potent and did not inhibit bacteria.

In relation to ciprofloxacin, the antibacterial activity of the extracts was low, but in comparison with the literature data on the activity of *L. stoechas* extracts, NADES, and supercritical extracts showed significant potency. It has been clearly demonstrated that using green solvents and techniques can achieve a more efficient and rational use of materials in a green manner without generating solvent waste, and ensure obtaining extracts rich in bioactive components with antioxidant and antimicrobial capacity. Taking into account the wide variety of natural compounds and the possibility of system customization, it would potentially hinder the development of bacterial resistance [42].

The approach investigated in this work allows for the selective extraction of components. Moreover, the obtained extracts were of different chemical profiles and could, therefore, be used for different applications. NADES extracts were rich in polar polyphenolic compounds which possess significant antioxidant and antimicrobial properties. The antimicrobial activity of the extracts makes them potential constituents in the production of cosmetic, health, food, and veterinary-related products. Due to their antioxidant activity, the NADES extracts can be used for attenuating oxidative stress processes that are in the pathogenesis of many diseases.

On the other hand, supercritical extracts were rich in non-polar terpenic compounds which have numerous confirmed biological properties and potential applications. Due to their aromatic properties, they are very important constituents in the fragrance and perfume industries. Oxygenated terpenes are a parameter of the quality of extracts because highly active functional groups are considered responsible for numerous biological properties, such as antimicrobial activity [43]. Therefore, the prevalence of oxygenated components in supercritical extracts makes them potentially good antimicrobial agents and of great importance in health, food, and cosmetic-related areas. Additionally, the identified terpenes are attributed to numerous medically important properties. Verbenone in vivo antidiabetic activity was reported together with the conclusion that it could reduce diabetes-associated complications such as cardiovascular disease, by enhancing lipids’ metabolism [44]. Fenchone exhibited an antidiarrheal effect [45], as well as a significant analgesic activity without inducing motor incoordination [46]. Borneol showed promising results as an effective adjuvant for improving drug delivery to the central nervous system [47], while Kong et al. [48]’s in vivo study determined that borneol possessed an anti-cerebral ischemia effect. Yang et al. [49] suggested the anti-inflammation potentials of bornyl acetate in patients with osteoarthritis, while viridiflorol exerted anti-mycobacterial, anti-inflammatory, and antioxidant activities [50].

In addition, the advantage of NADES extracts compared to the control extracts (ethanol and water) was confirmed and it can be seen in several aspects. Water was more efficient than ethanol for the extraction of polyphenols, but higher polyphenolic contents were achieved in NADES (Bet:EG) extracts. Although the higher antioxidant activity of water extracts was determined, it was not statistically different from Bet:EG, which exerted a more prominent antimicrobial activity. NADES extracts represent ready-to-use products, hence, additional purification steps are not required. Conversely, water extracts are susceptible to microbiological contamination and to preserve their stability, water removal is required representing an additional step that increases the process costs. Lastly, due to its low selectivity, water can extract non-bioactive components, thereby producing bioactive extracts of lower purity [51].

## 3. Materials and Methods

### 3.1. Material and Chemicals

*L. stoechas* L. ssp. *stoechas* flowers were commercial samples purchased in Celeiro, Lisbon, Portugal. Particle size reduction was performed using IKA Tube Mill 100 control (Staufen, Germany) at 20,000 rpm for 30 s. The mean particle size (0.31 ± 0.05 mm) of the material was determined using the vibration sieve sets (CISA, Cedaceria, Spain). The moisture content of the plant material (7.24 ± 0.18%) was determined with a moisture analyzer DAB (Kern, Balingen, Germany), equipped with a halogen quartz glass heater (400 W) and set at 105 °C.

Glycerol (99.5% purity) was purchased from Scharlau (Barcelona, Spain). Betaine (≥99% purity) and d-glucose monohydrate (≥97.5% purity) were purchased from Sigma-Aldrich (St. Louis, MO, USA). Ethylene glycol (≥99.5% purity) and ethanol (99% purity) were purchased from Carlo Erba (Val-de-Reuil, France).

### 3.2. Preparation and Characterization of NADES

NADES mixtures were prepared by mixing components in the adequate molar ratio and then heated (40 °C) and stirred until a clear liquid was formed. The NADESs and the molar ratio which were applied were betaine/ethylene glycol (Bet:EG) (1:3), betaine/glycerol (Bet:Gly) (1:2), and glycerol/glucose (Gly:Glu) (4:1). The water content of NADES was determined using Karl-Fischer titration using an 831 KF Coulometer with generator electrode (Metrohm, Herisau, Switzerland) and it was below 1% for all NADES. Measurements were conducted in triplicates.

### 3.3. Extraction Procedure

#### 3.3.1. Supercritical Carbon Dioxide Extraction

Supercritical CO_2_ extraction was carried out in a lab-scale apparatus with the following specifications: pneumatic pump (Williams P250V300), mass flow meter (Rheonik RHM 007), tubular extractor (316SS; 570 mm length, 24 mm I.D.; HiP), back-pressure regulator (Tescom Europe, model 26–1700, Selmsdorf, Germany), and separator (Swagelok 316L-HDF4-500). The extractions were performed using 30 g of plant material under the following conditions: pressure 200 bar, temperature 40 °C, extraction time 3 h, and CO_2_ flow 20 g/min (total amount of used CO_2_ 4470.87 ± 31.85 g). All experiments were performed in triplicates. The parameters of extraction were selected based on the previously conducted experimental work [21]. The obtained extracts were stored at 4 °C until further analysis.

#### 3.3.2. Ultrasound-Assisted Extraction with Deep Eutectic Solvents

Plant material after supercritical CO_2_ extraction (residue) and non-residue material (control) were used for ultrasound-assisted NADES extraction. The material was mixed with different NADES in a ratio of 1:20 (plant material:NADES; *w/w*). Ultrasound-assisted extractions were carried out at temperatures 30 and 60 °C, for 30 min, with an ultrasonic power of 100 W, and frequency of 50–60 Hz (Grant XUB5, UK). Extractions were conducted in cycles of 15 min, with stirring in a vortex for 1 min between cycles to speed up the extraction, according to Vieira et al. [52]. Flasks were positioned in the same position in the ultrasound bath during each extraction. After extraction, filtration was performed, and the obtained extracts were stored at 4 °C until further analyses. Extractions were performed in triplicates.

### 3.4. Gas Chromatography-Mass Spectrometry (GC-MS) Analysis

An Agilent 8890 gas chromatograph (Agilent Technologies, Palo Alto, CA, USA) connected to a mass spectrometer (series 5977E, Agilent Technologies, Palo Alto, CA, USA) was used for the analysis. The components of *L. stoechas* lipophilic extracts were separated on an HP-5MS capillary column (30 m × 0.25 mm, 0.25 µm, Agilent Technologies, Palo Alto, CA, USA). The injector temperature was set at 250 °C with the injected sample of 3 µL in a split mode of 1:50. Helium of 99.99% purity was used as a carrier gas in a constant flow regime of 1 mL/min. The following temperature program was set at 70 °C (2 min) which was increased by 3 °C/min to reach 200 °C and maintained at constant temperature for 15 min. The separated components were analyzed with mass spectrometry (70 eV) with a scanning *m*/*z* range of 30–300. The injector and detector temperatures were 250 °C and 300 °C, respectively. Qualitative identification of the compounds was performed using Wiley 9 (Wiley, New York, NY, USA) and NIST 17 (National Institute of Standards and Technology, Gaithersburg, MD, USA) mass spectral libraries as well as literature data of retention indices calculated with C9–C25 alkanes. The analysis of each sample was performed in three replicates and the results are expressed as mean data.

### 3.5. High-Performance Liquid Chromatography (HPLC)

Determination of polyphenolic components was performed by high-performance liquid chromatography (HPLC) method with UV detection, on Cosmosil 5C18-MS-II column (Nacalai Tesque, Inc., Kyoto, Japan), 250 mm long with an internal diameter of 4.6 mm, filled with 5 μm particles. The HPLC system used for the analysis (Agilent, 1260 Infinity II series) consisted of a quaternary pump (G7111A), a column chamber (G7116A), a photo-diode array detector (G7115A), an autosampler (G7157A), and a fraction collector (G1364E). The system was operated using the computer program Prep LC Online.

The separation of analyzed compounds was performed by using gradient elution for 50 min with 1% CH_3_COOH (in Milli-Q water) as phase A and methanol as phase B, with an initial ratio of A:B as 80:20. The gradient conditions were: 0–5 min 80% of A, 5–15 min 80–40% of A, 15–35 min holding 20% of A, 35–40 min 20–40% of A, 40–50 min 40–80% of A. During the analysis, the flow rate was 1.0 mL min^−1^, temperature at 25 °C, and detection at 250, 300, 320, and 360 nm. The injection volume of samples was 20 µL.

The standard stock solution for components (ellagic acid, ferulic acid, syringic acid, sinapic acid, caffeic acid, gallic acid, chlorogenic acid, neochlorogenic acid, cryptochlorogenic acid, rutin, myricetin, kaempferol-3-rutinoside, quercetin, luteolin, herniarin, coumarin) was prepared in methanol. The compound ellagic acid was calibrated at 250 nm, compounds ferulic acid, syringic acid, sinapic acid, and coumarin were calibrated at 300 nm, while caffeic acid and herniarin were detected at 320 nm and rutin at 360 nm. The calibration for ellagic acid was obtained at seven concentrations (5–100 mg/L), for caffeic acid at seven concentration (10–1000 mg/L), for ferulic acid at seven concentrations (10–500 mg/L), for syringic acid at eight concentrations (10–1000 mg/L), for sinapic acid at seven concentrations (10–500 mg/L), for rutin at seven concentration (10–500 mg/L), for herniarin at eight concentrations (10–200 mg/L) and for coumarin at eight concentrations (10–150 mg/L). The linearity of the calibration curve was confirmed by R^2^ = 0.99850 for caffeic acid, R^2^ = 0.99993 for syringic acid, R^2^ = 0.99922 for sinapic acid, R^2^ = 0.99975 for ferulic acid, R^2^ = 0.99665 for coumarin, R^2^ = 0.99842 for rutin, R^2^ = 0.99796 for ellagic acid, and R^2^ = 0.99991 for herniarin. The retention time for caffeic acid was 14.376 min, for syringic acid 14.500 min, for sinapic acid 17.300 min, for ferulic acid 17.500 min, for coumarin 18.085 min, for rutin 19.034 min, for ellagic acid 19.726 min, and for herniarin 19.931 min. The limit of detection (LOD) was as follows: caffeic acid 0.15 mg/L, syringic acid 0.71 mg/L, sinapic acid 0.45 mg/L, ferulic acid 0.34 mg/L, coumarin 0.62 mg/L, rutin 0.30 mg/L, ellagic acid 1.63 mg/L, and herniarin 1.40 mg/L. The limit of quantification (LOQ) was for caffeic acid 0.46 mg/L, syringic acid 2.17 mg/L, sinapic acid 1.37 mg/L, ferulic acid 1.03 mg/L, coumarin 1.88 mg/L, for rutin 0.91 mg/L, for ellagic acid 4.95 mg/L, and for herniarin 5.31 mg/L. The sample analysis was performed in duplicate, and two injections were performed from each prepared solution. The compounds contained in the analyzed samples were expressed in µg/mL of extract. Gallic acid, chlorogenic acid, neochlorogenic acid, cryptochlorogenic acid, myricetin, kaempferol -3-rutinoside, quercetin, and luteolin were not identified in the extracts.

### 3.6. Determination of Antioxidant Activity

#### 3.6.1. DPPH Assay

The antioxidant activity of extracts was analyzed using the 2,2-diphenyl-1-picrylhydrazyl (DPPH) assay [53]. Different volumes of extracts were mixed with 95% methanol solution and 90 μM DPPH solution. After the 60-min incubation period at room temperature, absorption was measured at a wavelength of 515 nm (6300 Spectrophotometer, Jenway, Dunmow, UK). The antioxidant activity was expressed as IC_50_ (mg/mL) value which represents the concentration of the extract which inhibits 50% DPPH radicals. All measurements were performed in triplicate.

#### 3.6.2. ABTS Assay

The 2,2′-azino-bis(3-ethylbenzothiazoline-6-sulfonic acid (ABTS) assay was performed according to Miller et al. [54] method. ABTS and potassium persulfate were dissolved in distilled water to a final concentration of 7 mM and 2.45 mM, respectively. These two solutions were mixed and the mixture was left at room temperature in a dark space for 12 h. The ABTS+ solution was diluted with distilled water to an absorbance of 0.700 ± 0.02 at 732 nm. Samples were added to diluted ABTS+ solution and the absorbance was measured at 3 min (732 nm) after mixing using a spectrophotometer (6300 Spectrophotometer, Jenway, Dunmow, UK). All measurements were performed in triplicates and the results were expressed as IC_50_ (mg/mL).

#### 3.6.3. FRAP Assay

The ferric ion-reducing antioxidant power (FRAP) of the samples was determined according to the assay based on the reduction of Fe^3+^ [55]. Different dilutions of liquid extract were mixed with phosphate buffer (0.2 M, pH 6.6) and 1% potassium ferricyanide in glass tubes. Tubes were incubated at 50 °C for 20 min. After incubation, 10% trichloroacetic acid solution was added to the reaction mixture. Tubes were then centrifuged at 3000 rpm for 10 min and supernatant was further mixed with double distilled water and 0.1% ferric chloride solution. Absorbance was measured at 700 nm (6300 Spectrophotometer, Jenway, Dunmow, UK). Antioxidant activity was further expressed as IC_50_ value (mg/mL), which causes a reduction of 50% Fe^3+^ ions. All experiments were performed in triplicates, and the results are expressed as mean values.

### 3.7. Determination of Antibacterial Activity

The determination of minimal inhibitory concentrations (MIC) of obtained extracts was performed by modified broth microdilution method in Mueller Hinton Broth (Fluka, BioChemica, Germany) according to Clinical Laboratory and Standard Institute (CLSI) M7-A7 document [56]. Bacteria were collected from different clinical samples obtained from the Microbiological Service of the Public Health Institute of Osijek-Baranja County, Croatia. *Bacillus subtilis*, *Staphylococcus aureus*, *Escherichia coli,* and *Pseudomonas aeruginosa* previously cultured on Mueller Hinton agar were suspended in saline at a density standardized to 0.5 McFarland standard turbidity and added to 100 μL of two-fold serially diluted obtained extracts. Each plate contained a growth control (bacterial inoculum without extracts), background control (broth and ethanol), and the antibacterial standard ciprofloxacin (Hospira, Hurley, Maidenhead, Berkshire, England, UK). After incubation at 37 °C for 24 h, another incubation at 37 °C for three hours was performed using triphenyltetrazolium chloride as a reducing agent indicator for microbial growth. The MIC was defined as the lowest extract concentration at which no color change due to microbial growth occurred, derived from triplicate analyses and normalized against the negative control.

### 3.8. Statistical Analysis

All analyses were carried out in triplicate and the results were expressed as means ± standard deviation (SD). Mean values were considered significantly different at a *p* < 0.05 confidence level, after the performance of the one-way ANOVA statistical analysis followed by Tukey’s HSD post hoc test. Pearson’s correlation coefficients (0.05 level; 2-tailed) were calculated in GraphPad Prism 8.0.1.

## 4. Conclusions

In this study, a sustainable procedure for using the plant resource *L. stoechas* was established. By combining the sequential application of supercritical CO_2_ and NADES with ultrasound-assisted extraction, the attainment of extracts rich in terpenes and polyphenols with significant antioxidant and antibacterial activity was achieved. Among NADES extracts, the highest polyphenolic content, as well as antioxidant activity, were determined in Bet:EG extracts. These extracts further exhibited significant antibacterial activity against bacteria, with the inhibitory activity being more pronounced against Gram-positive bacteria compared to Gram-negative. In comparison with conventional solvents, water, and ethanol, Bet:EG proved to be superior in terms of polyphenolic content as well as biological activities. Additionally, the supercritical extract, rich in oxygenated terpenes, also exhibited significant activity. Furthermore, the components identified in the obtained extracts also possess other confirmed biological properties and potential applications, hence these extracts can be used in other pharmaceutical, food, and cosmetic-related products. Due to their aromatic properties, they are very important constituents in the fragrance and perfume industries. The approach established in this study to obtain *L. stoechas* extracts represents the development of products in line with the principles of green and sustainable development and can successfully respond to the increased green consumerism. Extracts have the potential in the strategy of sustaining life and promoting good health, through roles in addressing AMR, as well as controlling foodborne diseases.

## Figures and Tables

**Table 1:** Antioxidant activity of extracts determined using DPPH, ABTS, and FRAP assays (expressed as IC_50_ values; mg/mL).

Solvent	Material	Temperature (°C)	DPPH-IC_50_	ABTS-IC_50_	FRAP-IC_50_
Betaine/ethylene glycol	residue	30	0.60 ± 0.02 ^h^	0.96 ± 0.13 ^ef^	1.14 ± 0.07 ^gh^
		60	0.36 ± 0.03 ^h^	0.58 ± 0.01 ^f^	0.60 ± 0.02 ^h^
	fresh	30	0.43 ± 0.04 ^h^	0.76 ± 0.02 ^ef^	0.79 ± 0.00 ^gh^
		60	0.35 ± 0.04 ^h^	0.50 ± 0.02 ^f^	0.70 ± 0.01 ^h^
Betaine/glycerol	residue	30	3.20 ± 0.23 ^b–e^	3.32 ± 0.19 ^b^	3.30 ± 0.14 ^cd^
		60	3.38 ± 0.52 ^bcd^	3.36 ± 0.02 ^b^	3.29 ± 0.07 ^cd^
	fresh	30	3.00 ± 0.05 ^c–f^	3.02 ± 0.00 ^b^	3.20 ± 0.05 ^cd^
		60	2.52 ± 0.29 ^d-g^	2.60 ± 0.11 ^bc^	3.18 ± 0.26 ^cd^
Glycerol/glucose	residue	30	2.35 ± 0.09 ^efg^	2.48 ± 0.04 ^bcd^	2.65 ± 0.03 ^de^
		60	1.81 ± 0.28 ^g^	1.73 ± 0.11 ^cde^	1.39 ± 0.15 ^fg^
	fresh	30	2.73 ± 0.11 ^def^	3.08 ± 0.10 ^b^	1.93 ± 0.18 ^f^
		60	2.13 ± 0.08 ^fg^	1.37 ± 0.07 ^def^	2.05 ± 0.03 ^ef^
Ethanol 96%	residue	30	3.72 ± 0.08 ^bc^	11.14 ± 1.56 ^a^	8.60 ± 0.88 ^a^
		60	3.98 ± 0.21 ^b^	3.43 ± 0.22 ^b^	4.50 ± 0.07 ^b^
	fresh	30	5.31 ± 0.99 ^a^	3.37 ± 0.12 ^b^	4.80 ± 0.09 ^b^
		60	3.29 ± 0.25 ^bcd^	3.25 ± 0.05 ^b^	3.77 ± 0.04 ^c^
Water	residue	30	0.25 ± 0.08 ^h^	0.51 ± 0.02 ^f^	0.63 ± 0.03 ^h^
		60	0.30 ± 0.03 ^h^	0.47 ± 0.03 ^f^	0.52 ± 0.04 ^h^
	fresh	30	0.47 ± 0.01 ^h^	0.62 ± 0.01 ^ef^	0.55 ± 0.02 ^h^
		60	0.43 ± 0.01 ^h^	0.50 ± 0.00 ^f^	0.72 ± 0.01 ^gh^
Supercritical extract			2.34 ± 0.21	1.73 ± 0.12	18.58 ± 3.09

Means followed by different letters differ significantly, based on Tukey’s HSD test at *p* < 0.05.

**Table 2:** Pearson’s correlation coefficient analysis between phenolic compounds and antioxidant activity in *L. stoechas* NADES extracts.

Assay	Total Phenol Content	Ellagic Acid	Caffeic Acid	Ferulic Acid	Syringic Acid	Sinapic Acid	Rutin	Herniarin	Coumarin
DPPH									
r	−0.7864	−0.5971	−0.6416	−0.4748	−0.6946	−0.8458	−0.6702	−0.6905	0.1582
R squared	0.6184	0.3566	0.4117	0.2254	0.4825	0.7154	0.4491	0.4768	0.02502
*p* (two-tailed)	<0.0001	0.0054	0.0023	0.0344	0.0007	<0.0001	0.0012	0.0008	0.5054
*p* value summary	****	**	**	*	***	****	**	***	ns ^1^
ABTS									
r	−0.6045	−0.438	−0.4378	−0.3491	−0.4967	−0.5783	−0.5568	−0.454	−0.00847
R squared	0.3654	0.1919	0.1917	0.1219	0.2467	0.3345	0.31	0.2061	7.17 × 10^−5^
*p* (two-tailed)	0.0048	0.0534	0.0535	0.1314	0.0259	0.0076	0.0108	0.0444	0.9717
*p*-value summary	**	ns	ns	ns	*	**	*	*	ns
FRAP									
r	−0.6791	−0.5246	−0.5101	−0.4028	−0.5691	−0.6743	−0.613	−0.5348	0.1185
R squared	0.4611	0.2752	0.2602	0.1623	0.3239	0.4547	0.3758	0.286	0.01405
*p* (two-tailed)	0.001	0.0176	0.0216	0.0782	0.0088	0.0011	0.0041	0.0151	0.6187
*p*-value summary	***	*	*	ns	**	**	**	*	ns

Correlation is significant at the 0.05 level (2-tailed); ^1^ ns—not significant. **** *p* < 0.0001; *** *p* ≤ 0.001; ** *p* < 0.01; * *p* < 0.05.

**Table 3:** Antibacterial activity of *L. stoechas* extracts expressed as minimum inhibitory concentrations (MIC) (mg·mL^−1^).

Solvent	Material	Temperature (°C)	Gram-Negative Bacteria		Gram-Positive Bacteria	
			*Escherichia coli*	*Pseudomonas* *aeruginosa*	*Bacillus subtilis*	*Staphylococcus* *aureus*
Betaine/ethylene glycol	residue	30	6.250	6.250	3.125	3.125
		60	3.125	3.125	1.563	1.563
	control	30	3.125	3.125	1.563	1.563
		60	1.563	1.563	0.781	0.781
Ethanol 96%	residue	30	3.125	3.125	1.563	1.563
		60	1.563	1.563	0.781	0.781
	control	30	3.125	3.125	1.563	1.563
		60	1.563	1.563	0.781	0.781
Water	residue	30	1.563	3.125	1.563	1.563
		60	1.563	3.125	1.563	1.563
	control	30	1.563	3.125	1.563	1.563
		60	1.563	3.125	1.563	1.563
Supercritical CO_2_	200 bar/40 °C/20 g/L		3.39	6.775	3.39	3.39
Control ciprofloxacin *			3.13	7.89	1.56	3.13

* µg/mL.

## Data Availability

Not applicable.

## Supplementary Materials

The following supporting information can be downloaded at: https://www.mdpi.com/article/10.3390/antibiotics12061031/s1, Table S1: HPLC analysis of *Lavandula stoechas* extracts obtained with NADES. Results are expressed in µg/mL.
