# Supplementary material for: Towards a Greener Approach for Biomass Valorization: Integration of Supercritical Fluid and Deep Eutectic Solvents
Source: Antibiotics (Basel). 2023 Jun 8;12(6):1031. doi: 10.3390/antibiotics12061031 (PMC10295627; doi:10.3390/antibiotics12061031)
Supplement: Supplementary file 1 [file antibiotics-12-01031-s001.zip › antibiotics-2407739-supplementary.pdf]

Supplementary material

Table S1. HPLC analysis of *Lavandula stoechas* extracts obtained with NADES. Results are expressed in µg/mL.

Solvent	Material	Temperature (°C)	Ellagic acid	Caffeic acid	Ferulic acid	Syringic acid	Sinapic acid	Rutin	Herniarin	Coumarin
Betaine/ethylene glycol	residue	30	13.29 ± 1.01^{d}	6.29 ± 0.99^{f}	14.32 ± 1.55^{c}	12.62 ± 1.68^{e}	34.37 ± 1.44^{c}	234.97 ± 5.81^{d}	79.61 ± 4.84^{c}	0.27 ± 0.12^{fg}
		60	42.21 ± 2.63^{a}	4.75 ± 0.40^{g}	27.84 ± 0.60^{a}	12.58 ± 0.79^{e}	39.46 ± 1.89^{b}	438.93 ± 4.60^{a}	97.86 ± 1.86^{b}	0.59 ± 0.06^{d}
	control	30	33.52 ± 2.71^{b}	3.47 ± 0.26^{h}	13.58 ± 0.58^{c}	8.52 ± 0.44^{f}	59.25 ± 3.68^{a}	355.34 ± 5.68^{b}	81.35 ± 2.09^{c}	0.40 ± 0.00^{e}
		60	19.01 ± 0.41^{c}	9.58 ± 0.18^{e}	16.06 ± 0.21^{b}	12.67 ± 0.43^{e}	33.92 ± 1.92^{c}	348.37 ± 11.92^{b}	43.40 ± 0.57^{e}	0.77 ± 0.01^{c}
Betaine/glycerol	residue	30	10.88 ± 0.31^{def}	0.00 ± 0.00^{k}	2.42 ± 0.10$^{f\text{-}j}$	0.00 ± 0.00^{h}	0.00 ± 0.00^{d}	72.89 ± 0.71^{ij}	8.20 ± 0.27^{ij}	0.24 ± 0.01$^{f\text{-}i}$
		60	12.24 ± 0.69^{de}	1.96 ± 0.03^{ij}	2.82 ± 0.09^{fgh}	0.00 ± 0.00^{h}	0.00 ± 0.00^{d}	85.23 ± 3.00^{ghi}	32.71 ± 0.29^{f}	0.12 ± 0.01^{i}
	control	30	10.73 ± 0.44^{def}	0.00 ± 0.00^{k}	2.94 ± 0.33^{efg}	0.00 ± 0.00^{h}	0.00 ± 0.00^{d}	78.17 ± 1.57^{hij}	18.17 ± 1.12^{h}	0.26 ± 0.00^{fgh}
		60	8.97 ± 0.46^{fgh}	1.70 ± 0.04^{ij}	3.36 ± 0.29^{def}	0.00 ± 0.00^{h}	0.00 ± 0.00^{d}	93.29 ± 1.20^{g}	9.35 ± 0.12^{ij}	0.27 ± 0.01^{fg}
Glycerol/glucose	residue	30	7.97 ± 0.07^{fgh}	0.00 ± 0.00^{k}	2.62 ± 0.19$^{f\text{-}i}$	0.00 ± 0.00^{h}	0.00 ± 0.00^{d}	85.53 ± 1.30^{ghi}	6.05 ± 0.09^{j}	0.26 ± 0.02^{fgh}
		60	13.50 ± 0.96^{d}	1.26 ± 0.31^{j}	4.17 ± 0.21^{de}	0.00 ± 0.00^{h}	0.00 ± 0.00^{d}	110.02 ± 5.75^{ef}	31.10 ± 0.44^{fg}	0.12 ± 0.01^{i}
	control	30	9.49 ± 0.66e^{fg}	0.00 ± 0.00^{k}	3.22 ± 0.30^{def}	0.00 ± 0.00^{h}	0.00 ± 0.00^{d}	88.53 ± 3.97^{gh}	5.63 ± 0.19^{j}	0.30 ± 0.00^{efg}
		60	10.75 ± 1.10^{def}	2.11 ± 0.07^{ij}	4.36 ± 0.17^{d}	0.00 ± 0.00^{h}	0.00 ± 0.00^{d}	120.36 ± 1.31^{e}	7.46 ± 0.07^{j}	0.31 ± 0.01^{ef}
Ethanol	residue	30	6.59± 0.03^{gh}	1.72 ± 0.00^{ij}	1.25 ± 0.04^{jk}l	0.00 ± 0.00^{h}	0.00 ± 0.00^{d}	0.00 ± 0.00^{l}	19.92 ± 0.05^{h}	0.14 ± 0.01^{hi}
		60	7.15 ± 0.24^{gh}	2.41 ± 0.26^{i}	1.12 ± 0.11^{jkl}	4.75 ± 0.32^{g}	0.00 ± 0.00^{d}	68.54 ± 2.75^{jk}	21.28 ± 0.07^{h}	0.13 ± 0.04^{i}
	control	30	6.04 ± 0.10^{h}	0.00 ± 0.00^{k}	0.00 ± 0.00^{l}	0.00 ± 0.00^{h}	0.00 ± 0.00^{d}	55.53 ± 1.99k	12.52 ± 0.27^{i}	0.42 ± 0.02^{e}
		60	7.79 ± 0.03^{fgh}	1.55 ± 0.06^{ij}	1.48 ± 0.10^{ijk}	4.06 ± 0.04^{g}	0.00 ± 0.00^{d}	72.48 ± 1.94^{ij}	29.71 ± 0.22^{fg}	0.19 ± 0.01^{ghi}
Water	residue	30	10.39 ± 0.43^{def}	40.64 ± 0.16^{a}	1.64 ± 0.12$^{g\text{-}k}$	33.92 ± 1.19^{c}	32.68 ± 0.16c	96.72 ± 8.66^{fg}	55.01 ± 0.11^{d}	1.06 ± 0.00^{a}
		60	16.93 ± 1.02c	23.72 ± 0.62c	0.95 ± 0.06^{kl}	28.07 ± 2.15^{d}	33.08 ± 0.13^{c}	257.48 ± 1.09^{c}	128.67 ± 1.71^{a}	0.90 ± 0.03^{b}
	control	30	13.16 ± 1.14^{d}	28.09 ± 0.06^{b}	1.52 ± 0.11$^{h\text{-}k}$	47.81 ± 2.71^{a}	33.03 ± 0.17^{c}	67.19 ± 0.71^{jk}	27.48 ± 2.66^{g}	1.09 ± 0.09^{a}
		60	12.90 ± 0.12^{d}	20.82 ± 0.06^{d}	0.82 ± 0.04^{kl}	39.75 ± 0.32^{b}	31.78 ± 0.19^{c}	118.54 ± 0.75^{e}	52.48 ± 2.66^{d}	0.85 ± 0.00^{bc}

Data are expressed as the mean ± standard deviation (n = 20). Different letters (a–h) in the same column indicate statistical differences at the 0.05 ($p < 0.05$).
